# Supplementary material for: Response surface optimization for cadmium biosorption onto the pre-treated biomass of red algae Digenia simplex as a sustainable indigenous biosorbent
Source: PeerJ. 2025 Aug 4;13:e19776. doi: 10.7717/peerj.19776 (PMC12330821; doi:10.7717/peerj.19776)
Supplement: Supplemental Information 2 [file peerj-13-19776-s002.pdf]

Smp_1080

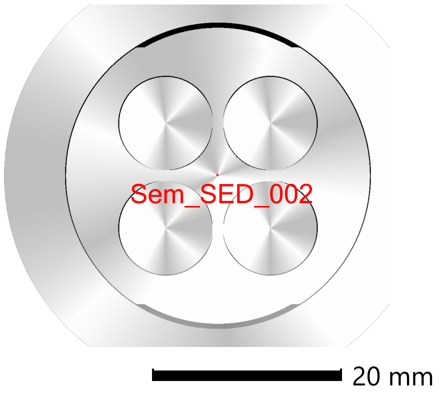

Sem_SED_002

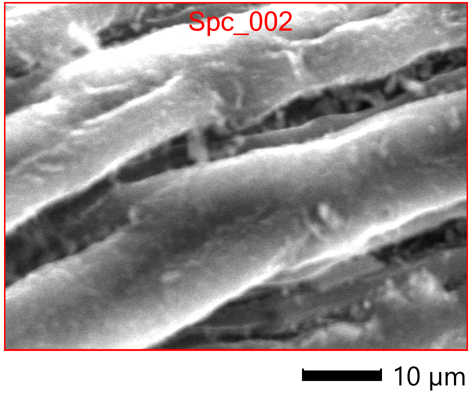

Signal SED
Landing Voltage 30.0 kV
WD 10.1 mm
Magnification x2,200
Vacuum Mode HighVacuum

Items	Value
measurement conditions	
Acceleration voltage	30.00 kV
Probe current	0.00 nA
Magnification	x 2200
Process time	T2
Measurement detector	First
Live time	30.00 seconds
Real time	30.94 seconds
Dead time	3.00
Count rate	7701.00 CPS

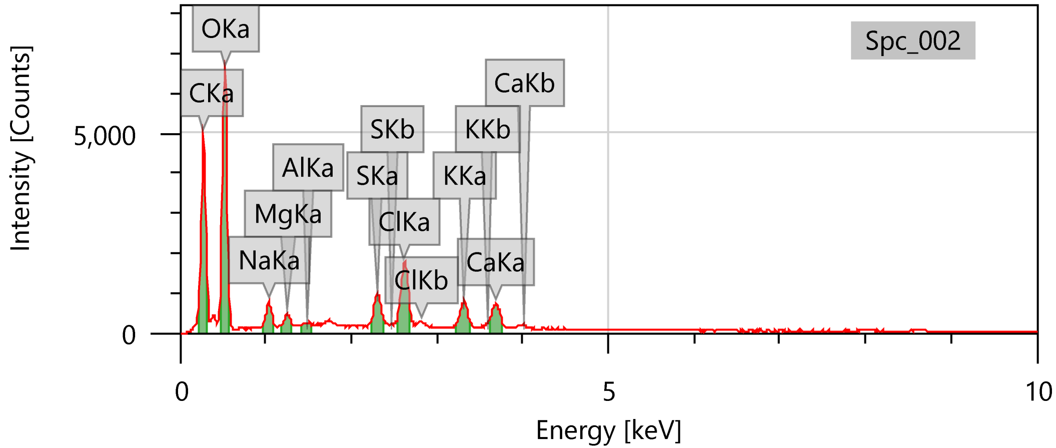

Display name	Standard data	Quantification method	Result Type
Spc_002	Standardless	ZAF	Metal

Element	Line	Mass%	Atom%
C	K	40.55±0.13	48.93±0.16
O	K	52.89±0.25	47.91±0.23
Na	K	1.85±0.04	1.17±0.02
Mg	K	0.49±0.02	0.29±0.01
Al	K	0.08±0.01	0.05±0.00
S	K	0.81±0.01	0.37±0.01
Cl	K	1.72±0.02	0.70±0.01
K	K	0.81±0.01	0.30±0.00
Ca	K	0.79±0.01	0.28±0.00
Total		100.00	100.00
Spc_002			Fitting ratio 0.0196

Smp_1080

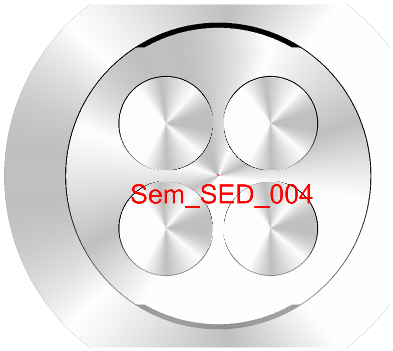

Sem_SED_004

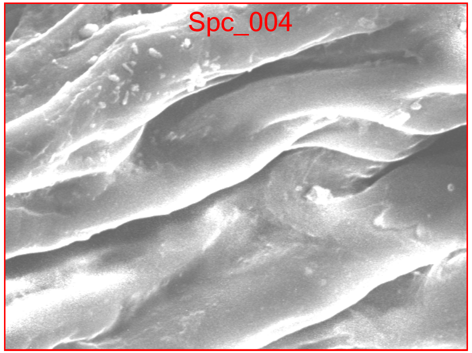

Signal SED
Landing Voltage 30.0 kV
WD 10.1 mm
Magnification x3,300
Vacuum Mode HighVacuum

Items	Value
measurement conditions	
Acceleration voltage	30.00 kV
Probe current	0.00 nA
Magnification	x 3300
Process time	T2
Measurement detector	First
Live time	30.00 seconds
Real time	30.65 seconds
Dead time	2.00
Count rate	4239.00 CPS

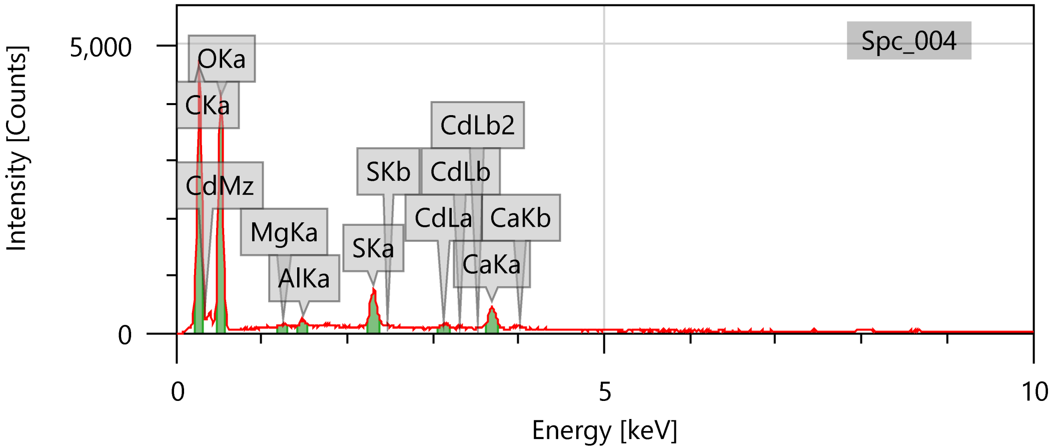

Display name	Standard data	Quantification method	Result Type
Spc_004	Standardless	ZAF	Metal

Element	Line	Mass%	Atom%
C	K	45.33±0.15	53.11±0.17
O	K	52.29±0.32	45.99±0.28
Mg	K	0.09±0.01	0.05±0.01
Al	K	0.20±0.01	0.10±0.01
S	K	1.01±0.02	0.44±0.01
Ca	K	0.74±0.02	0.26±0.01
Cd	L	0.34±0.02	0.04±0.00
Total		100.00	100.00
Spc_004			Fitting ratio 0.0254
