# Supplementary figures and images for: Response surface optimization for cadmium biosorption onto the pre-treated biomass of red algae Digenia simplex as a sustainable indigenous biosorbent
Source: PeerJ. 2025 Aug 4;13:e19776. doi: 10.7717/peerj.19776 (PMC12330821; doi:10.7717/peerj.19776)

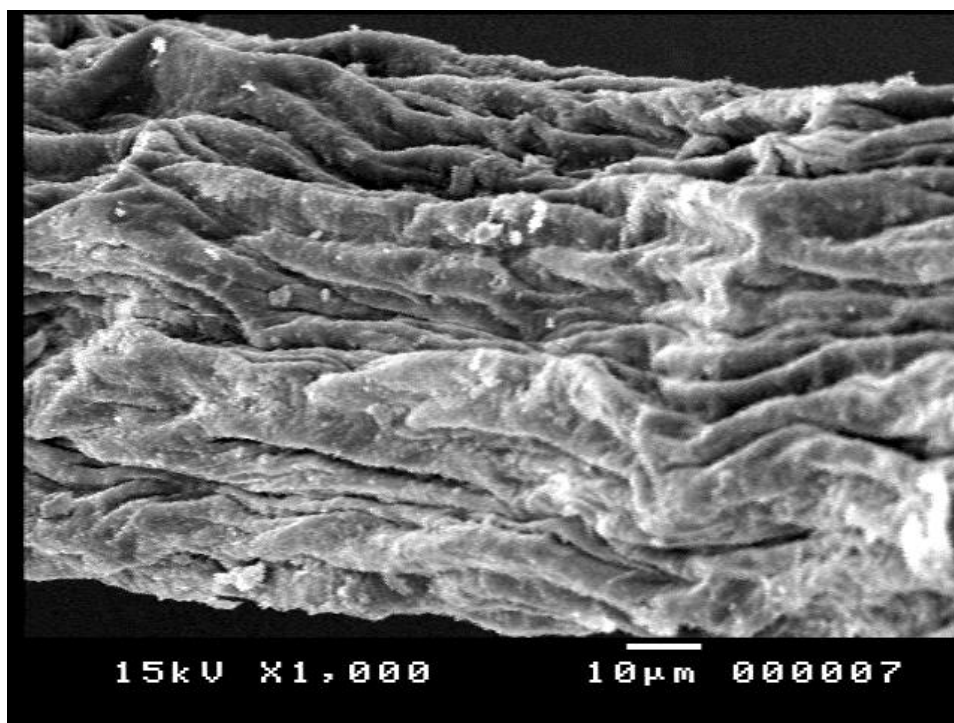

A) Before Cd biosorption

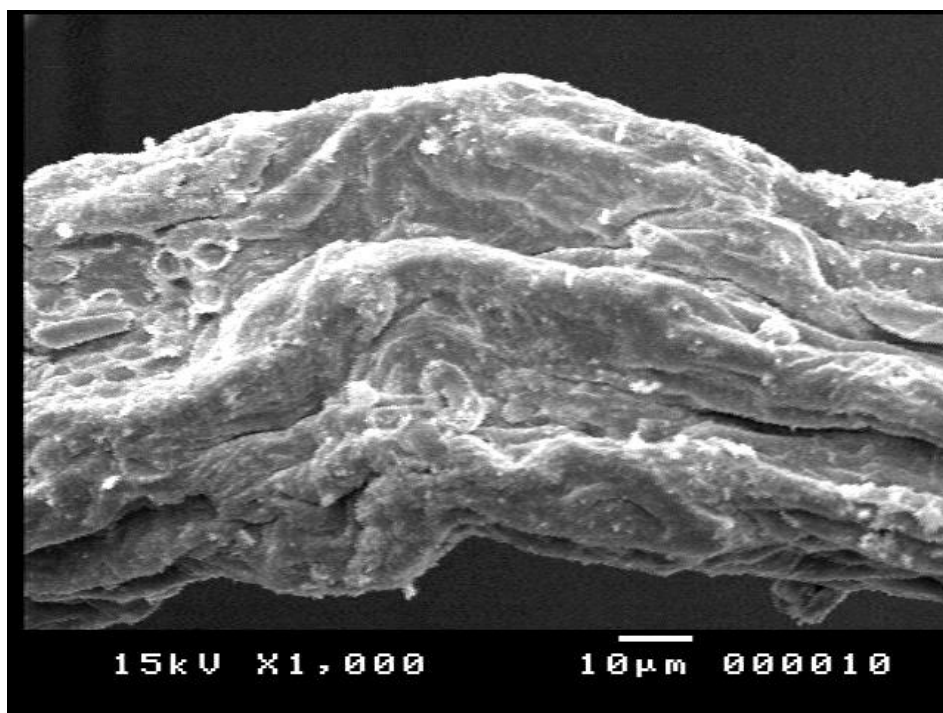

B) After Cd biosorption

Supplement: Supplemental Information 5 [file peerj-13-19776-s005.pdf]
