# Supplementary material for: Response surface optimization for cadmium biosorption onto the pre-treated biomass of red algae Digenia simplex as a sustainable indigenous biosorbent
Source: PeerJ. 2025 Aug 4;13:e19776. doi: 10.7717/peerj.19776 (PMC12330821; doi:10.7717/peerj.19776)
Supplement: Supplemental Information 6 [file peerj-13-19776-s006.pdf]

Response surface optimization for cadmium biosorption onto the pre-treated biomass of red algae *Digenia simplex* as a sustainable indigenous biosorbent

Sedky H. A. Hassan[1], Maryam M. Alomran[2]*, Nada I. A. Alsugiran[2], Mostafa Koutb[3], Hassan Ahmed[4] and Mustafa A. Fawzy[4, 5]

[1]Department of Biology, College of Science, Sultan Qaboos University, Muscat 123, Oman

[2]Department of Biology, College of Science, Princess Nourah bint Abdulrahman University, P.O. Box 84428, Riyadh 11671, Saudi Arabia

[3]Department of Biology, Faculty of Science, Umm Al-Qura University, Makkah 24381, Saudi Arabia

[4]Biology Department, Faculty of Science, Taif University, P.O. Box 11099, Taif 21944, Saudi Arabia

[5]Botany and Microbiology Department, Faculty of Science, Assiut University, Assiut 71516, Egypt

*Correspondence: mmalomran@pnu.edu.sa

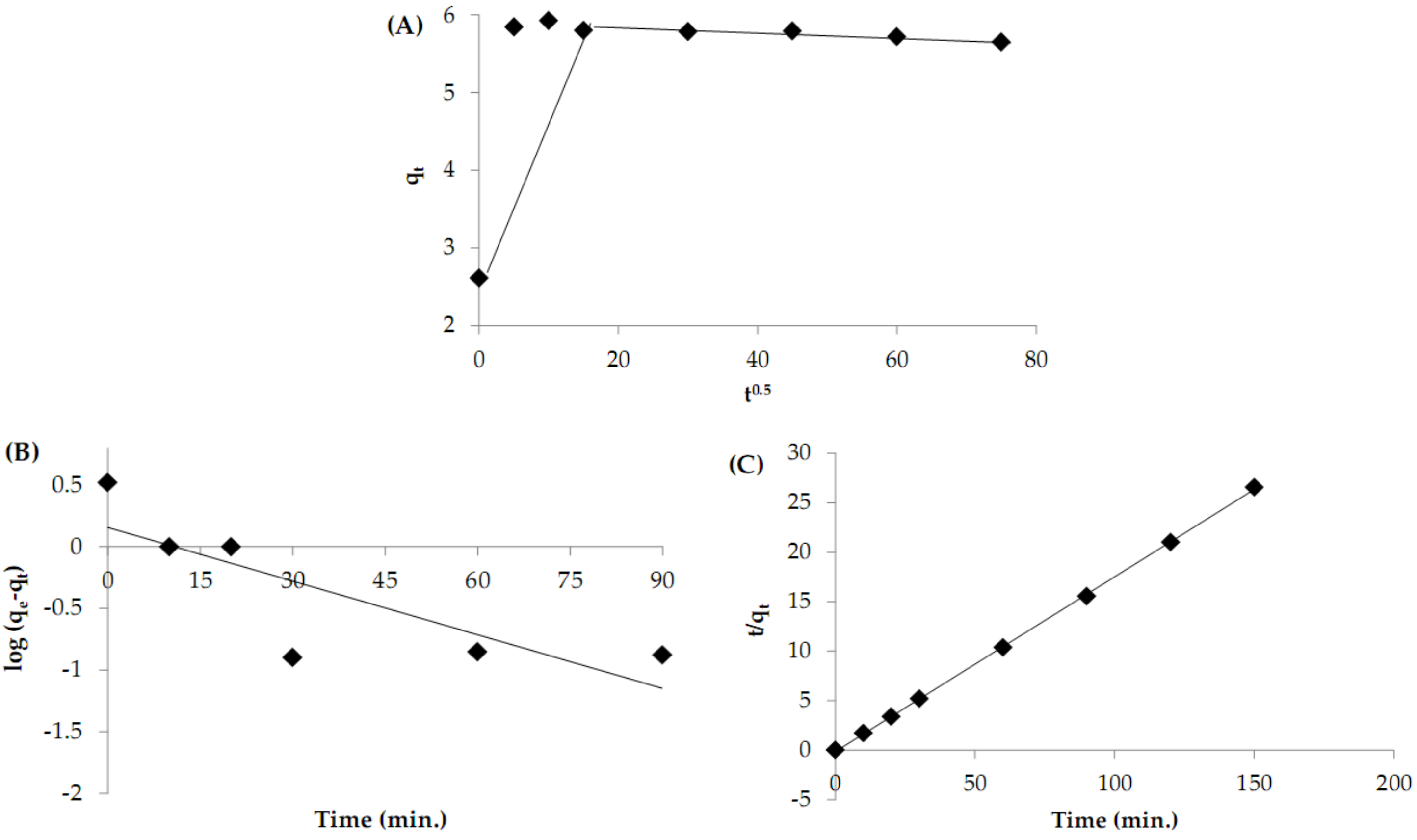

Figure S1. Graphs of A) intra-particle, B) pseudo-first-order, and C) pseudo-second-order kinetic models for Cd^{2+} ions biosorption on DSC biosorbent.

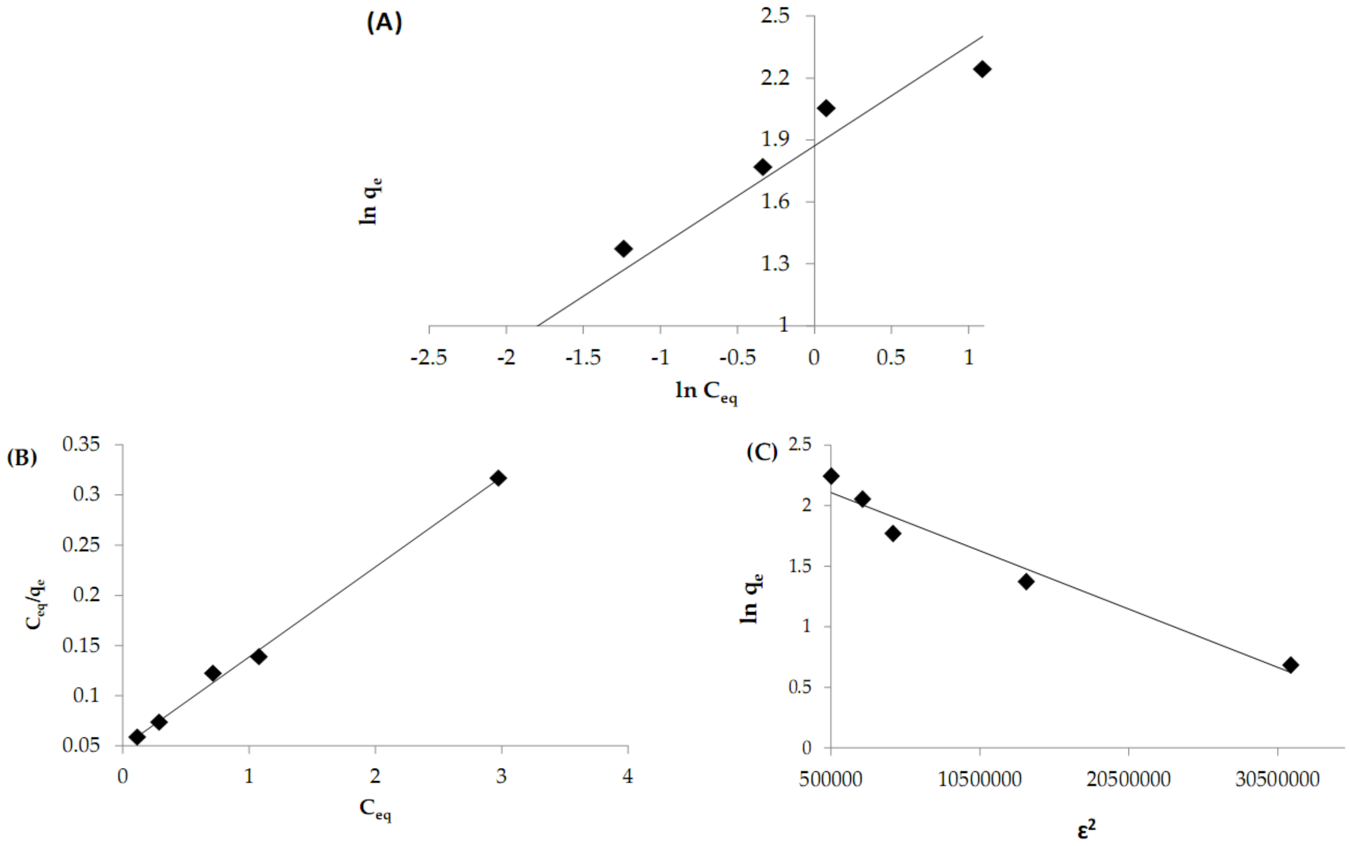

Figure S2. Graphs of A) Freundlich, B) Langmuir, and C) D–R adsorption isotherms models for Cd^{2+} ions biosorption on DSC biosorbent.

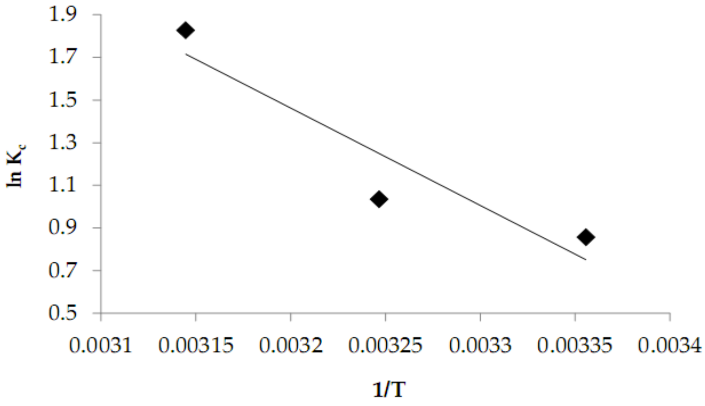

Figure S3. Plot of lnKc versus 1/T for Cd^{2+} ions biosorption on DSC biosorbent.
